# Isolation and characterization of *Stenotrophomonas rhizophila* T3E: a multifunctional rhizobacterium enhancing tomato growth and soil health

**DOI:** 10.3389/fpls.2025.1692957

**Published:** 2025-10-23

**Authors:** Xiancui Zhang, Haoran Liao, Peiwen Cai, Tong Cai, Hongkai Si, Jinfeng Yuan, Yanan Yu, Mingxing Zhao, Peng Li, Xianduo Zhang, Lu Jiang, Liang Yang, Choufei Wu

**Affiliations:** ^1^ School of Life Science, Huzhou University, Huzhou, China; ^2^ Shandong Center for Disease Control and Prevention, Jinan, China; ^3^ Institute of Sericulture, Huzhou Academy of Agricultural Sciences, Huzhou, China; ^4^ Laboratory of Invertebrate Pathology, College of Animal Sciences, Zhejiang University, Hangzhou, China

**Keywords:** *Stenotrophomonas rhizophila*, PGPR, tomato, colonization, soil fertility

## Abstract

**Introduction:**

Plant growth-promoting rhizobacteria (PGPR) serve as sustainable alternatives to chemical fertilizers and pesticides for improving crop productivity. Among them, *Stenotrophomonas rhizophila* has demonstrated considerable potential to enhance plant growth, yet the mechanisms underlying its effects on plant development and soil health remain insufficiently elucidated.

**Methods:**

In this study, *S. rhizophila* strain T3E was isolated and identified from the tomato (*Solanum lycopersicum*) rhizosphere based on morphological and phylogenetic analyses. The growth-promoting traits of T3E were comprehensively characterized through germination and pot experiments, integrated with phytohormone profiling, gene expression and biochemical analyses, soil assessment, and whole-genome sequencing.

**Results:**

Inoculation with T3E significantly enhanced radicle length, primary root length, plant height, and seedling fresh weight, while quantitative assays revealed increased levels of indole-3-acetic acid (IAA) and abscisic acid (ABA) in the roots. qRT-PCR analysis showed that T3E upregulated multiple root growth-related genes (e.g., *EAT2*, *LAX2*, *GTS1*, *GRFs*) and T3E colonization-related genes (*pyrB*, *flmH*, *pilR*, *bopD*), supporting its strong root colonization ability. Physiologically, T3E treatment enhanced SOD activity, increased glycine betaine and soluble sugar levels, and reduced MDA content, suggesting improved root health and stress resistance. Whole-genome sequencing revealed a 4.31 Mb circular chromosome with 3,744 coding sequences, diverse secondary metabolite biosynthetic gene clusters, and abundant carbohydrate-active enzyme (CAZyme) genes. Soil assays demonstrated that T3E inoculation improved key physicochemical properties (TN, TP, TK, AK) and significantly enhanced the activities of soil enzymes, such as phosphatase, CAT, urease, and sucrase.

**Discussion:**

These findings identify *S. rhizophila* T3E as a promising multifunctional PGPR that promotes plant growth, strengthens rhizosphere colonization, and enhances soil fertility. This study provides a solid theoretical basis for developing microbial biofertilizers and advancing sustainable agricultural practices.

## Introduction

Tomato (*Solanum lycopersicum*) is one of the most important vegetable crops worldwide and has long served as a classic model system for studies of plant development and physiology (Barringer, 2004; Zhao et al., 2022). However, global climate change, the extensive use of chemical pesticides, and highly intensive agricultural production systems have led to soil quality degradation, exposing tomato plants to a variety of biotic and abiotic stresses that severely impair growth and yield, resulting in substantial economic losses (Weiskopf et al., 2020; Fiodor et al., 2021; Giordano et al., 2021). Consequently, there is an urgent need for environmentally friendly and cost−effective approaches to sustainable agricultural production. In this regard, the application of plant growth−promoting rhizobacteria (PGPR) as biological inoculants holds great promise for agriculture, forestry, and environmental remediation (Kunal et al., 2023; Chattaraj et al., 2025).

Rhizosphere microorganisms, which are microbial communities residing in plant root systems, play a pivotal role in promoting plant health, enhancing stress resistance, and improving soil ecosystem functions (Favela, 2021; Li et al., 2022). These microorganisms are classified into beneficial (2-5%), harmful (8-15%), and neutral (80-90%) groups (Khoso et al., 2024; Feng et al., 2025). Among them, plant growth−promoting rhizobacteria (PGPR) constitute the beneficial fraction of the rhizosphere microbiota, encompassing genera such as *Pseudomonas*, *Enterobacter*, *Bacillus*, and *Burkholderia* (Etesami and Beattie, 2018). Studies have demonstrated that PGPR directly stimulate plant growth by secreting plant hormones-such as gibberellins and auxins-and volatile organic compounds (Hou et al., 2021). For example, *Pseudomonas aeruginosa* HMR1 can produce indole−3−acetic acid (IAA), significantly enhancing plant growth (Bhojiya et al., 2022). Furthermore, the PGPR *Sinomonas gamaensis* NEAU−HV1 induces the initiation of lateral root primordia (LRP) through the canonical auxin signaling cascade, thereby promoting the formation of additional lateral roots (LRs) (Fu et al., 2025). Simultaneously, PGPR can indirectly support plant growth under adverse conditions such as drought and salinity via biological nitrogen fixation, phosphate solubilization, potassium mobilization, and enhancement of plant immune responses (De Kesel et al., 2021). Ambust et al. reported that *Pseudomonas* sp. SA3 produces biosurfactants capable of degrading organic compounds and increasing the hydrophobicity of petroleum in contaminated soils, thereby enhancing plant resilience to pollutants, alleviating stress during growth and development, and improving overall growth performance (Bhojiya et al., 2022).

*Stenotrophomonas* is a genus of Gram−negative bacteria belonging to the phylum Bacteroidetes, widely distributed in soil, water bodies, and plant rhizospheres (Shih et al., 2022). Studies have shown that certain *Stenotrophomonas* strains play pivotal roles in agriculture, industry, and medicine-particularly in regulating plant growth, aiding bioremediation, synthesizing industrial enzymes, and combating multidrug−resistant pathogens (Singh and Jha, 2017; Youseif, 2018; Niu et al., 2023). For example, *S. maltophilia* LGMB417 produces siderophores and degrades organic substrates such as starch and cellulose, thereby facilitating plant nutrient uptake (Ercole et al., 2021). In another case, following infection of the wheat rhizosphere by *Fusarium pseudograminearum* (Fp), *S. rhizophila* SR80 became significantly enriched in the rhizosphere (3.7 × 10^7^ cells/ g); reintroduction of SR80 into soil subsequently promoted both above and below−ground growth of wheat (Liu et al., 2021). Furthermore, *S.rhizophila* DSM14405T harbors a diffusible signal factor (DSF)−mediated quorum−sensing system, endowing it with strong salt-alkali tolerance and robust root−colonization ability (Liu et al., 2022). Recent studies further demonstrated that the type VI secretion system(T6SS) of *S. rhizophila* CFBP13503 suppresses the transmission of the plant pathogen *Xanthomonas campestris* pv. campestris 8004 from seeds to seedlings, thereby promoting radish seed germination (Garin et al., 2024). Comparable transmission dynamics have been documented during the germination of rapeseed (*Brassica napus*) and common bean (*Phaseolus vulgaris*) seeds (Garin et al., 2025). Comprehensive comparative genomic analyses reveal that PGPR characteristics are widespread in *S. rhizophila.* For instance, the presence of genes involved in siderophore production and phosphate solubilization, as well as biosynthetic pathways for IAA, polyamines and cytokinins, endows this species with strong plant growth–promoting potential (Zhao et al., 2024; Chauviat et al., 2025). Despite these advances, the application prospects and underlying regulatory mechanisms of *Stenotrophomonas* in plant growth promotion remain to be fully elucidated.

Efficient colonization of the rhizosphere by probiotic bacteria is a prerequisite for their plant−beneficial functions. In the present study, we first isolated a strain of *S. rhizophila* from tomato rhizosphere soil and evaluated its growth−promoting effects as well as its capacities to synthesize IAA and abscisic acid (ABA). We then performed whole−genome sequencing to elucidate its genomic features and employed RT-qPCR to quantify the expression of colonization−related genes. Moreover, we specifically aimed to determine whether *S. rhizophila* could regulate tomato root growth by modulating root development-related gene expression and enhancing antioxidant defense mechanisms. Finally, after reintroducing the *S. rhizophila* strain into soil, we assessed changes in plant growth promotion, soil physicochemical properties and key enzyme−activity indices. This work aims to provide theoretical guidance for the development of rhizosphere−based microbial fertilizers and to promote sustainable agricultural production.

## Materials and methods

### Isolation and identification of the T3E strain

Tomato rhizosphere soil was harvested from tomato field in Zhejiang province, China (120.13°N 30.87°W). Rhizosphere soils were collected as described previously (McPherson et al., 2018). The roots were lightly cleaned to eliminate non-adherent soil, leaving an approximately 1-mm coating of soil on their surfaces(n=15). Approximately 1,500 bacterial were isolated from the rhizosphere soil and purified by plate culture with LB medium (Hopebio, Qingdao, China). Based on preliminary screening for plant growth-promoting traits, one strain, designated T3E (*S. rhizophila*), was selected for further characterization and functional analyses. The strain was purified, and genomic DNA was extracted using a bacterial genomic DNA extraction kit (Qiagen DNeasy kit, catalog number 69506). The 16S rRNA gene was amplified with the universal primers 27F (5′-AGAGTTTGATCCTGGCTCAG-3′) and 1492R (5′-GGTTACCTTGTTACGACTT-3′). The PCR reaction mixture (25 μL) contained: 12.5 μL of 2×Taq Master Mix, 1 μL of each upstream and downstream primer (10 μmol/L), 1 μL of DNA template, and 9.5 μL of ddH2O. The PCR conditions were as follows: 95°C for 5 minutes; 94°C for 30 seconds, 55°C for 30 seconds, and 72°C for 90 seconds for 35 cycles; followed by a final extension at 72°C for 10 minutes. After confirming the PCR products by electrophoresis on a 1.5% agarose gel, the samples were sent to Wuhan Qingke Biotechnology Co., Ltd. for sequencing. A phylogenetic tree was constructed using the Neighbor-Joining method in MEGA 11 software. The 16S rRNA gene sequences were compared with those from public databases and aligned with the 16S rRNA sequence of the T3E strain to determine its phylogenetic position. Strain T3E was deposited in the China General Microbiological Culture Collection Center (CGMCC, Beijing) as CGMCC No.35226.

For scanning electron microscopy (SEM) analysis, T3E was initially fixed in 2.5% glutaraldehyde, washed three times with PBS, and subsequently post-fixed in 1% osmium tetroxide. 1 mL of the fermented culture of the T3E strain was collected by centrifugation, and the cells were placed into 1.5 mL centrifuge tubes containing 3% glutaraldehyde fixation solution for fixation for 24 hours. After fixation, the samples were washed three times with PBS and then post-fixed with osmium tetroxide. The samples were then dehydrated through a graded ethanol series (50%, 70%, 80%, 95%, 100%) for 15 min and embedded in epoxy resin (Dr. Spurr’s kit, Electron Microscopy Sciences, Hatfield, PA). Ultrathin sections were stained with 1% uranyl acetate and examined using SEM transmission electron microscope (Hitachi SU8000, Tokyo, Japan) at an accelerating voltage of 80 kV. The samples were dehydrated through an ethanol gradient (50%, 70%, 80%, 90%, and 100%) and then vacuum-dried using an EM CPD 300 critical point dryer (Leica, Wetzlar, Germany). After samples were coated with gold using a JEOL JFC-110E Ion Sputter, they were observed under a scanning electron microscope (Merlin, Zeiss, Germany).

### Verification of the growth-promoting ability of the T3E strain

The tomato variety Sweetheart No. 8 was selected based on its superior pomological traits, which are highly esteemed by consumers in China. Tomato seeds were obtained from Yinong Agriculture Co., Ltd. (Zhejiang, China). The seeds underwent surface sterilization using the following procedure: immersion in 70% ethanol (Sangon, Shanghai, China) for 1 minute, followed by treatment with 10% sodium hypochlorite (Sangon, Shanghai, China) for 5 minutes, and then subjected to five consecutive rinses with sterile distilled water. To evaluate the growth-promoting potential of T3E, both seed germination and pot assays were conducted. For the seed germination assay, the sterilized seeds were subsequently treated with sterile T3E fermentation supernatant. To obtain the T3E supernatant, the strain was initially streaked onto LB medium, and individual colonies were transferred to LB liquid medium for 24 hours of shaking culture at 30 °C and 180 rpm. After culture, the bacterial suspension was centrifuged at 10,000 g for 5 minutes, and the supernatant was filtered through a 0.22 μm membrane filter (Millipore, Germany). The tomato seeds were then immersed in the filtered supernatant for 30 minutes (T3E group), while the control seeds were soaked in sterile LB liquid medium (CK group). After treatment, the seeds were placed on sterile, moist filter paper in Petri dishes and incubated at 27°C for 5 days. The radicle length (n=15) was then measured as the distance from the seed base to the root tip. For the pot assay, surface-sterilized tomato seeds were germinated on sterile moist filter paper for 5 days without exposure to the T3E fermentation supernatant. The resulting seedlings were transplanted into 10 cm × 8.5 cm pots filled with approximately 150 g of sterilized soil. Root irrigation was applied once with 10 mL per plant of a T3E bacterial suspension (1 × 10^7^ CFU/mL), which was prepared by centrifuging the culture to collect the cell pellet and resuspending it in sterile water. Control plants received an equal volume of sterile water. Thereafter, all plants were maintained under normal growth conditions with routine management in a climate-controlled sterile growth chamber (Percival growth chamber; CLF Plant Climatics) at 22°C/18°C (day/night) under a 16 h/8 h photoperiod. After 20 days, plant height and root length were measured manually using a ruler. The seedling fresh weight were measured with a precision balance (n=5). Except for the seed germination assay, all subsequent experiments described in this study were conducted in pot systems, where tomato seedlings were inoculated by root irrigation with a T3E bacterial suspension (1×10^7^ CFU/mL), while control plants received sterile water.

### IAA, ABA content and growth phenotype analysis

Tomato rhizosphere soil, cultivated under standard conditions, was irrigated with a T3E bacterial suspension (1×10^7^ CFU/mL), with an equal volume of sterile water used as a control. After 10 days, the 0.2 g fresh root sample was extracted with 2 mL of phosphate buffer (pH 7.4) and then centrifuged at 1000 g for 10 minutes. The contents of IAA and ABA of tomato root were measured via enzyme-linked immunosorbent assay (ELISA) according to the protocols (ELISA kits BH100 and BH102, Zoonbio Biotechnology, Nanjing, China) (n=3).

### Measurement of enzyme activity and growth response gene of tomato root

Roots were collected from tomato seedlings grown in the pot assay system described above at 10 days post-inoculation (1×10^7^ CFU/mL, root irrigation) (n=3). The activities of superoxide dismutase (SOD), peroxidase (POD), malondialdehyde (MDA), glycine betaine and soluble sugars concent were measured in root after 10 days of T3E treatment. The enzyme activities of were measured by homogenizing a 0.5 g sample in 1 mL of PBS solution. The homogenate was then centrifuged at 8000 rpm for 10 minutes at 4 °C. SOD and POD activities were determined with a commercially available SOD kit and POD kit (Sangon Biotech, Shanghai, China), according to the instructions. Glycine betaine content was determined using a kit (Keming Biotechnology Co. Ltd.). Proline and soluble sugar contents were quantified with proline protein and soluble kits (Nanjing Institute of Bioengineering, Nanjing, China).

Quantitative real-time PCR (qRT-PCR) was performed to detect the relative expression of plant growth-related genes, including Target of early activation tagged (EAT) 2(*EAT2*), APETALA2-like protein (*AP2a*), LAX2 protein (*LAX2*), 4-coumarate-CoA ligase-like 1(*4CL*), WD repeat-containing protein GTS1(*GTS1*) and growth-regulating factor 4-like isoform X1(*GRFs*) (Lee et al., 1995; Lee and Cooksey, 2000; Gachomo et al., 2014; Lian et al., 2021; Lu et al., 2021; Jia et al., 2024). Healthy tomato root (without T3E) and T3E-inoculated root (1×10^7^ CFU/mL) were collected at 10 d post-inoculation in soil. Total RNA was extracted from 1g of soil samples by RNA PowerSoil Total RNA Isolation Kit (Mo Bio, USA), following manufacturers’ instructions. The RNA concentration and quality were measured using a NanoDrop (Biodropsis BD–2000), and the RNA from all samples was then adjusted to the same concentration. RNA of the desired quality (1 μg) was reverse transcribed into cDNA using HiScript™ II Q RT SuperMix (R223-01, Vazyme Biotech Co., Ltd.). growth‐related genes of root were determined at 10 d using RT-qPCR. The primers used in the qPCR assays are listed in **Supplementary Table 1**. All qPCR runs were conducted using SYBR qPCR Master Mix (Vazyme Biotech, Nanjing, China) and a Roche LightCycler 480 system (Roche, Basel, Switzerland), with sterile water included as a negative control. The thermocycler conditions were set as follows: an initial denaturation at 95°C for 5 min, followed by 40 cycles of 95°C for 10s, 60°C for 10s, and 72°C for 20s, with a final melting curve step (from 65°C to 92°C, at a rate of 0.5°C/s). The 2^−ΔΔct^ method was used to analyze the relative transcript levels of the genes of interest (n= 3).

### Genome sequencing, assembly, and annotation of T3E

The T3E strain was inoculated into LB liquid medium and cultured at 30°C with shaking at 180 rpm for 16–20 hours. Then, the bacterial suspension was centrifuged for 3 min at 6000 × g and the bacterial pellet was collected. Genomic DNA was extracted using DNA extraction kit (Qiagen, Hilden, Germany). The sample was subsequently sent to Majorbio Bio-pharm Technology Co., Ltd. (Shanghai, China) for sequencing using the PacBio RSII platform. The coding DNA sequence of the T3E genome was predicted using Glimmer v3.02 (http://ccb.jhu.edu/software/glimmer/index.shtml) and tandem repeats were identified using Tandem Repeats Finder (http://tandem.bu.edu/trf/trf.html) (Benson, 1999). Both rRNA and tRNA genes were predicted using Barrnap v0.8 and tRNAscan-SE v2.0, respectively. Functional annotation and classification were performed using BLAST and HMMER against five databases: non-redundant (NR), Pfam, Clusters of Orthologous Groups (COGs), Gene Ontology (GO), and the Kyoto Encyclopedia of Genes and Genomes (KEGG). To assess the secondary metabolic potential of T3E, the number of predicted biosynthetic gene clusters was estimated using antiSMASH 6.6.0rcl and BLAST (Blin et al., 2017). Moreover, the CAZyme repertoire was investigated and annotated through reference to the CAZy database (http://www.cazy.org/). The raw sequencing data, including both single-end and paired-end reads, are available in GenBank under BioProject ID PRJNA1242732.

### Quantitative real-time PCR validation of the T3E colonization gene

Pot experiments were performed as described above. To investigate T3E colonization in the rhizosphere of *S. lycopersicum*, pot experiments were conducted. Healthy tomato rhizosphere soil (without T3E) and T3E-inoculated soil (1×10^7^ CFU/mL) were collected at 0, 1, 5, 10 and 15 days post-inoculation. An equal amount of sterile distilled water was used to treat the tomato rhizosphere soil, as a negative control (n=3). The expression levels of colonization-related genes [aspartate carbamoyltransferase (gene0978, *pyrB*), short chain dehydrogenase/reductase family oxidoreductase (gene0061, *flmH*), two-component response regulator PilR(gene0155, *pilR*), and sugar-binding transcriptional regulator, LacI family (gene0059, *bopD*) were determined at 0, 1, 5, 10, and 15 days using RT-qPCR (n = 5)(Lee et al., 2000; Creti et al., 2006; Ziegler et al., 2006; Yang et al., 2023; Chen and Liu, 2024b). The primers used in the qPCR assays are listed in **Supplementary Table 1**. Total RNA was prepared and qPCR experiments performed as described above.

### Determination of soil physicochemical properties and enzyme activities

For soil assays, tomato seedlings were cultivated in pots and irrigated with T3E suspension (1×10^7^ CFU/mL) or sterile water. After 20 days, rhizosphere soil samples were collected and analyzed for physicochemical properties and soil enzyme activities following standard protocols(n=5). Soil samples were collected using a sterile dry brush to gently scrape the surface soil from the roots of control and T3E-treated tomato plants into sterile, self-sealing bags. The determination of soil physicochemical properties was performed following the standards outlined in the Soil Agricultural Chemical Analysis guidelines. Soil pH was measured using a pH meter on an appropriate amount of air-dried soil (sieved through a 20-mesh sieve), which was suspended in a 1:2.5 soil-to-water ratio. The soil bulk density was determined by weighing a 100 cm³ soil core after drying the sample at 40°C in the laboratory. To determine the Organic matter (OM) and total ammonium nitrogen (TN) concentration, the sample was centrifuged at 4,000 g for 10 minutes, and the supernatant was subsequently filtered through a 0.45 μm pore size regenerated cellulose syringe filter. The OM and TN were measured using the wet oxidation redox titration and micro-Kjeldahl methods, respectively. Ammonium and nitrate were analyzed by a continuous-flow analyzer. The available phosphorus (AP) was measured using the molybdenum blue method. In addition, we measured the total phosphorus (TP), total potassium (TK), available phosphorus (AP), alkaline nitrogen (AN), and available potassium (AK), following the method (Bao, 2000; Nichols and Wright, 2006). Briefly, TP content was analyzed using the sodium hydroxide fusion-molybdenum-antimony resistance colorimetric method, while TK was quantified by flame photometry (Flame Photometer 410, Sherwood). For the determination of AN, fresh soil (sieved through a 10-mesh sieve) was extracted with 0.01 mol/L KCl solution, filtered, and analyzed using a continuous flow analyzer. AP was extracted from air-dried soil (sieved through a 20-mesh sieve) using 0.5 M sodium bicarbonate (NaHCO_3_) and quantified using the molybdenum blue colorimetric method. AK content was measured by extracting air-dried soil (sieved through a 20-mesh sieve) with 1 M ammonium acetate, followed by flame photometry.

The activities of several soil enzymes were assessed, including phosphatase, peroxidase (POD), amylase, protease, catalase (CAT), glutamic acid decarboxylase (GAD), urease and sucrase. All soil enzyme activities were measured with a commercial activity assay kit (Solebao, Beijing, China) according to the manufacturer’s instructions(n=5).

### Statistical analysis

Statistical analyses were carried out using GraphPad Prism (version 9.0) and Statistical Package for the Social Sciences, version 20.0 (SPSS, Chicago, IL, USA). All the results are presented as the mean ± standard error (SE). Parametric/non-parametric statistical analyses were performed after the data were checked for normality and homogeneity of variance. Student’s t test was performed to compare the root length, IAA and ABA contents, gene expression changes, soil physical properties and enzyme activities between the experimental and control groups. In each case, the type of test is stated before the *P* value, and *P* ≤ 0.05 was considered statistically significant. All experiments were repeated at least three times in independent experiments. The software used included GraphPad Prism 9.0 and Python and R packages, run as standalone software on Windows 10.

## Results

### Characteristics of T3E isolated from tomato rhizosphere soil

**Figure 1:**
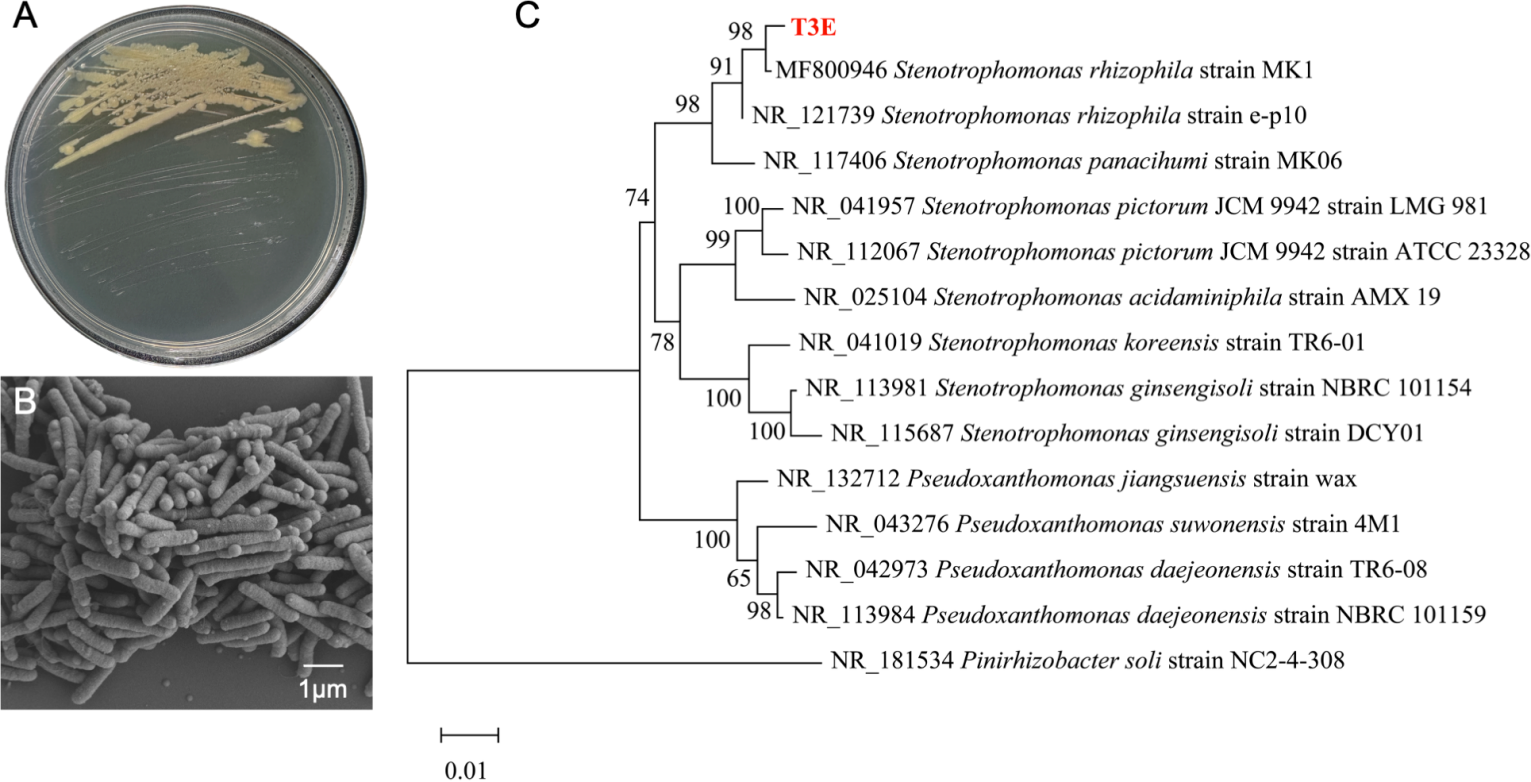
Phylogenetic analysis and morphological characteristics of *S. rhizophila* T3E strain. **(A)** Colonies of *S. rhizophila* on LB medium, showing smooth and moist appearance. **(B)** Scanning electron microscope (SEM) image showing rod-shaped bacterial cells with a scale bar of 1 μm. **(C)** Phylogenetic tree based on 16S rRNA gene sequences, demonstrating the close relationship of T3E with *S. rhizophila* MK1.

The T3E strain forms smooth, moist, round colonies with neat edges that are easily picked (**Figure 1A**). On solid media, the colonies appear pale yellow. The cells of this bacterium are about 1-2 μm in diameter and occur in singly or clusters (**Figure 1B**). SEM micrographs show clearly the production of outer membrane vesicles (OMVs) on the surface of bacteria (**Figure 1B**). The 16S rRNA gene sequence of the T3E strain shared 98% similarity with those of the *S. rhizophila* strain MK1 (MF800946), and a constructed phylogenetic tree demonstrated the closest phylogenetic relation-ship between T3E and Stenotrophomonas (**Figure 1C**).

### Plant growth-promoting effect of T3E

**Figure 2:**
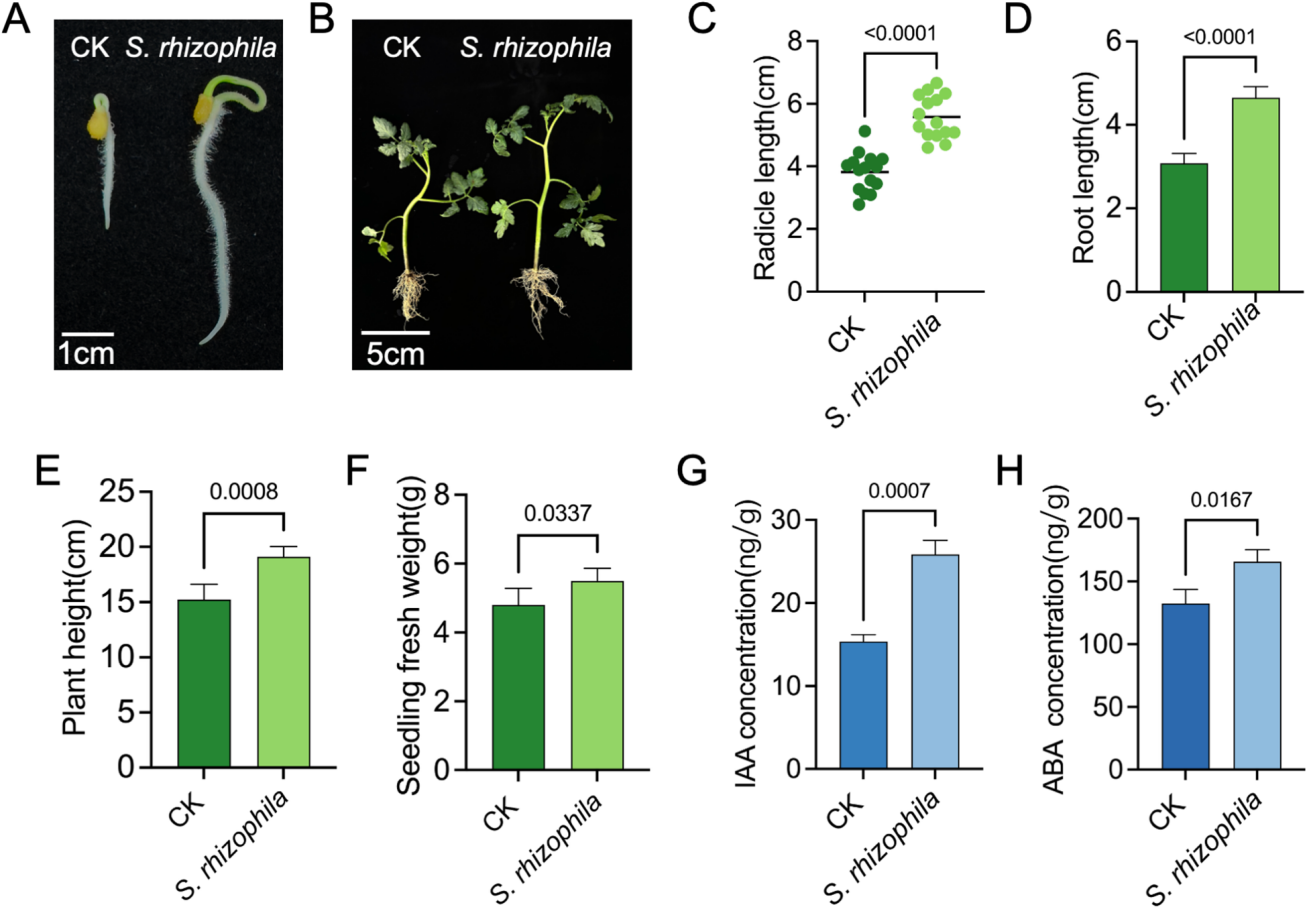
Growth-promoting effects of *S. rhizophila* T3E on tomato seedlings. **(A)** Tomato root development after 5 days of seedling growth following soil inoculation with T3E (scale bar = 1 cm). **(B)** Whole-seedling growth after 20 days, showing enhanced development compared with the control (CK), with a scale bar of 5 cm. The **(C)** radicle length, **(D)** root length, **(E)** plant height, and **(F)** seedling fresh weight was subsequently measured. **(G)** Higher indole-3-acetic acid (IAA) and abscisic acid (ABA) **(H)**concentrations in T3E-treated roots. The error bars indicate the ± SDs of the means. Statistical significance was determined by two-sided Student’s t-test.

Tomato seed treatment with T3E increased the seed germination and seedling growth compared to the control treatments (**Figures 2A, B**). Inoculation with the strain *S. rhizophila* T3E significantly enhanced tomato radicle length (**Figure 2C**), primary root length (**Figure 2D**), plant height (**Figure 2E**) and whole-seedling fresh weight (**Figure 2F**) (*p* < 0.05). For example, plant height was 19.12 ± 0.91 cm in T3E-treated seedlings versus 15.25 ± 1.37 cm in the control (CK) group (t=5.263, df=8, *p* = 0.0008).

Quantitative analysis of IAA and ABA demonstrated that T3E treatment markedly induced the biosynthesis of both hormones in tomato roots. Specifically, IAA levels in the control and T3E−treated groups were 15.36 ± 0.84 ng/g and 25.83 ± 1.71 ng/g, respectively (t=9.541, df=4, *p* = 0.0007), while ABA levels were 132.50 ± 11.08 ng/g and 165.80 ± 9.46 ng/g, respectively (t=3.960, df=4, *p* = 0.0167) (**Figures 2G, H**).

### Enzyme activity and growth response gene of tomato root

**Figure 3:**
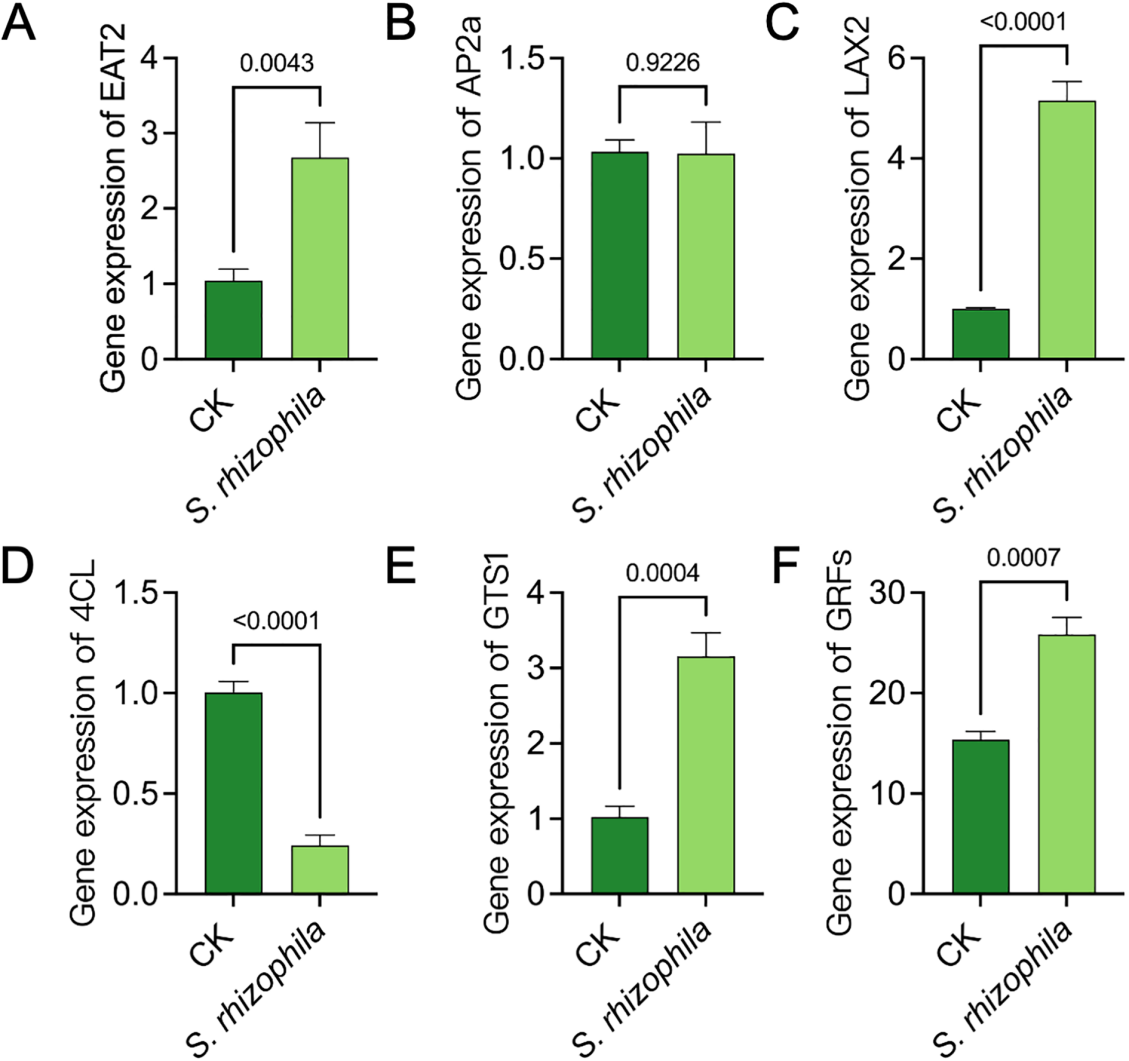
Gene expression analysis of root growth-related genes in tomato seedlings treated with *S. rhizophila* T3E. **(A)** *EAT2*, **(B)** *AP2a*, **(C)** *LAX2*, **(D)** *4CL*, **(E)** *GTS1*, **(F)** *GRFs*. The error bars indicate the ± SDs of the means. Statistical significance was determined by two-sided Student’s t-test.

To demonstrate whether the T3E regulate the expression of growth-related genes of tomato root, we detected the expression of the genes *EAT2*, *AP2a*, *LAX2*, *4CL*, *GTS1* and *GRFs* by qRT‐PCR (**Figure 3**). Interestingly, inoculation with *S. rhizophila* led to significant upregulation in the transcription levels of several growth-related genes including *EAT2*, *LAX2*, *GTS1*, and *GRFs*, compared to the uninoculated control(*p*<0.05). In contrast, *AP2a* showed no significant change(*p* = 0.9226), while *4CL* expression was significantly downregulated(*p*<0.0001**) (** **Figure 3**).

**Figure 4:**
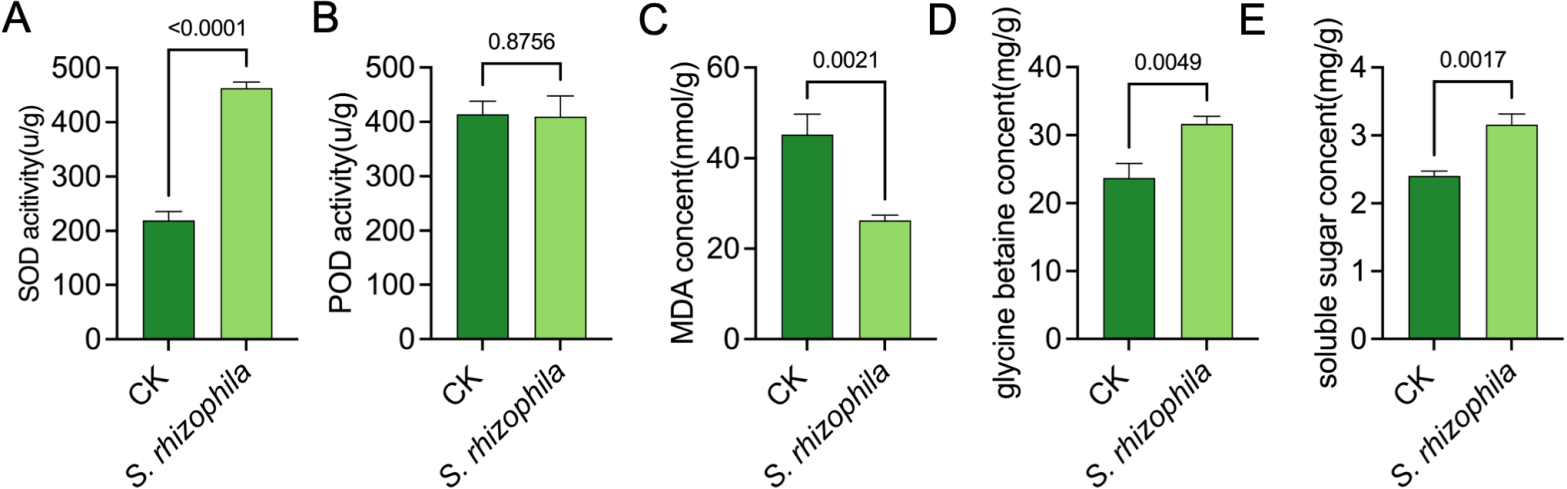
Physiological responses of tomato roots to *S. rhizophila* T3E treatment. **(A)** Superoxide dismutase (SOD) activity, **(B)** peroxidase (POD) activity, **(C)** Decreased malondialdehyde (MDA) content, **(D)** glycine betaine concentration, **(E)** Soluble sugar content. The error bars indicate the ± SDs of the means. Statistical significance was determined by two-sided Student’s t-test.

To characterise the physiological changes, we measured the activity of enzymes SOD and POD acitivity, the content of MDA, glycine betaine and soluble sugar in tomato root after 10 d of T3E treatment (**Figure 4**). The result showed that the activity of SOD in tomato root inoculated with T3E were observably increased compared to the control (462.70 ± 11.15 u/g vs. 219.30 ± 16.26 u/g; t=21.38, df=4, *p*<0.0001) (**Figure 4A**). Notably, there was no significant difference in POD activity (**Figure 3B**). In addition, the level of MDA was significantly lower in tomato root compared with the CK group, indicating a weak degree of cell damage (**Figure 4C**). The content of glycine betaine and soluble sugar were higher inoculated with T3E than in the control (**Figures 4D, E**).

### General features of the T3E genome

**Figure 5:**
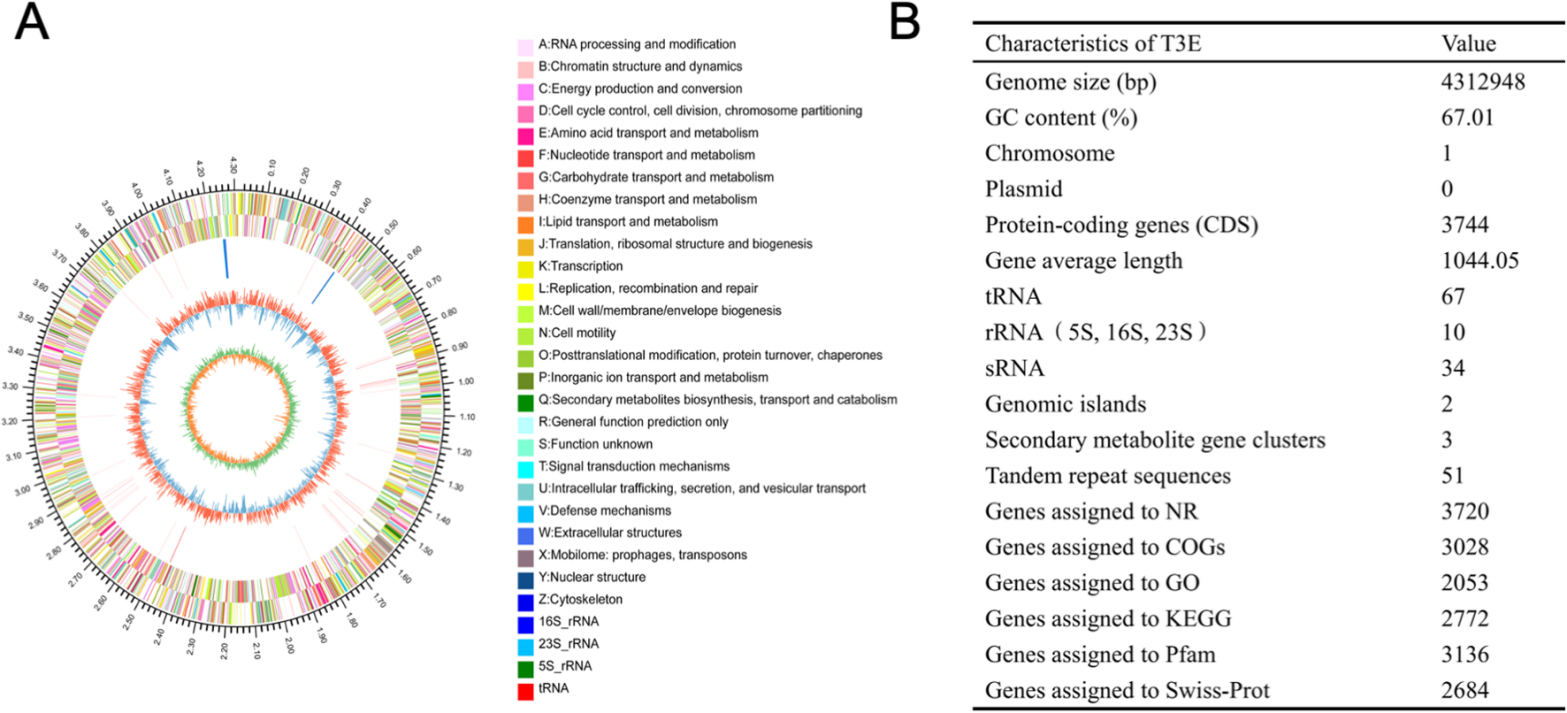
Genomic characteristics of *S. rhizophila* T3E strain. **(A)** Circular genome map of T3E. The outermost circle represents the entire genome size. The second and third circles display protein-coding sequences on the + and – strands, with distinct colors representing different COG functional categories. The fourth circle shows the locations of rRNA and tRNA sequences. The fifth circle illustrates the GC content, where red segments correspond to regions with higher GC content than the genome average, while blue segments indicate lower GC content; the peak height reflects the degree of deviation from the average. The sixth circle represents the GC skew (G–C/G + C), where a positive value suggests that CDS transcription is more likely to occur on the positive strand, and a negative value suggests transcription from the negative strand. **(B)** Genome annotation summary, including functional genes, tRNA, rRNA, and secondary metabolite biosynthesis clusters.

**Figure 6:**
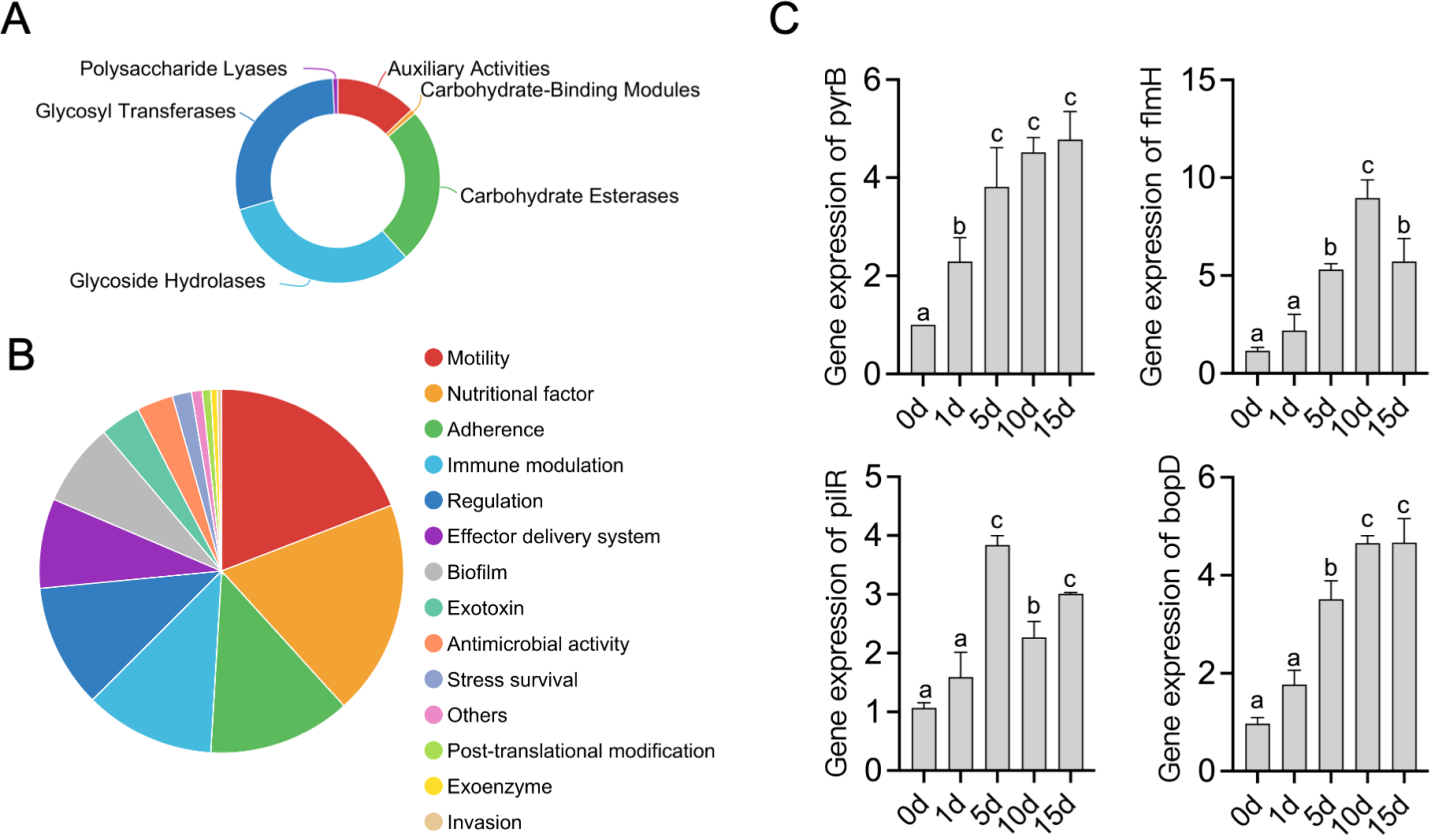
Functional analysis of *S. rhizophila* T3E genes. **(A)** Distribution of carbohydrate-active enzyme (CAZyme) families in the T3E genome. **(B)** Gene ontology (GO) categories for genes related to nutritional factors, motility, biofilm formation, and immune modulation in T3E. **(C)** Expression profiles of colonization-related genes (*pyrB*, *flmH*, *pilR*, *bopD*) over 15 days in the rhizosphere. Statistical significance was determined via one-way ANOVA with Tukey’s *post hoc* test. Different letters represent statistically significant differences (*P* < 0.05, n = 5).

In total, the *de novo* assembly of the sequences successfully generated a 4,312,948 bp circular chromosome with an average gene length of 1044.05 bp (**Figure 5**). One chromosomal region was identified as a prophage locus (**Figure 5A**). A total of 3,744 predicted coding sequences (CDSs), 67 tRNA genes, 10 rRNA operons, and 34 sRNA elements were identified in the genome of T3E. The T3E genome exhibits a GC content of 67.01% and an average gene length of 1,044.05 bp. A total of 3,720 coding sequences (CDSs) were annotated in the NR database, 3,028 in COGs, 2,053 in GO, 2,772 in KEGG, 3,136 in Pfam, and 2,684 in Swiss-Prot (**Figure 5B**). Three secondary metabolite biosynthetic were identified on the chromosome, corresponding to RiPP-like, class II lanthipeptide, and arylpolyene types (**Table 1**). A total of 125 genes related to carbohydrate-active enzymes were annotated, including members of 6 CAZy families, comprising 40 glycoside hydrolases, 36 glycosyl transferases, 31 carbohydrate esterases, 16 auxiliary activities, 1 carbohydrate-binding modules and 1 polysaccharide lyases (**Figure 6A**). In particular, T3E strains produced GH family enzymes that degraded cellulose, such as GH3, GH5 and GH74. The hemicellulose hydrolases produced by T3E primarily include GH2 and GH43 families. In addition, laccase-like multi-copper (AA1) and lignin-modifying peroxidases (AA2), which are responsible for the degradation of lignin, were detected in T3E strains. Additionally, T3E detected the GH18, GH19, and GH20 families responsible for chitin degradation (**Supplementary Table S2**). The analysis showed that T3E strains produce a wide range of CAZymes involved in polysaccharide degradation, suggesting their ability to efficiently acquire carbon and energy from the environment.

**Table 1:** Secondary metabolite biosynthetic gene clusters (BGCs) were predicted in T3E.

Location	Cluster ID	Type	Start	End	Similar cluster	Similarity	Gene no.
Chromosome	Cluster 1	RiPP-like	1443704	1454583	–	–	10
Chromosome	Cluster 2	lanthipeptide-class-ii	2297787	2352463	fuscachelin	44%	47
Chromosome	Cluster 3	arylpolyene	4096067	4139682	APE Vf	45%	37

The bacterial secretion system of T3E was checked based on the Protein export (map03070) pathway, and 45 key gene distributed in this pathway were identified (**Supplementary Figure 1**). A comprehensive genome-wide exploration of signal sequences, lipoprotein motifs, and other potential host cell components or binding motifs yielded an additional 528 putative surface-exposed proteins that may be associated with early colonization stages or virulence. We identified several genes (523) related to nutritional/metabolic factor, motility, adherence, immune evasion, regulation, effector delivery system, biofilm, exotoxin and antimicrobial activity/competitive advantage, some of which have been previously identified as colonization-related genes, whereas the other 5 genes showed no or vague functional predictions. And, the product of these genes are critical factors for host colonization (**Figure 6B**). RT–qPCR was utilized to determine how the response of T3E gene expression after colonization of the host gut. The expression of genes encoding nutritional/metabolic factor (*pyrB*), motility (*flmH*), adherence (*pilR*) and biofilm (*bopD*) proteins was measured based on their colonization-related functions (**Figure 6C**). T3E inoculation elicited significant upregulation of the colonization-related genes *pyrB*, *flmH*, *pilR*, and *bopD* (0-15d)(**Figure 6C**). In particular, compared with the level observed at 0 d (CK), the expression of pyrB (5-fold), flmH (8-fold), pilR (4-fold) and bopD (5-fold) reached extremely significant levels at 15 days (t=20.51, df=4, *p*<0.0001), 10 days (t=14.28, df=4, *p*<0.0001), 5 days (t=26.79, df=4, *p*<0.0001) and 10 days (t=32.57, df=4, *p*<0.0001), respectively (**Figure 6C**).

T3E was found to have K+ uptake transporter proteins genes, including the potassium-transporting ATPase subunit system (kdp), two pore domain potassium channel family protein, and the potassium transport system gene (kup) (**Supplementary Table S3**). Whole genome sequencing analysis shows that T3E synthesizes IAA from tryptophan. The vital genes for tryptophan biosynthesis, including the YerC/YecD family TrpR-related protein and indole-3-glycerol phosphate synthase TrpC, were present in T3E. In addition, the indole pyruvate ferredoxin oxidoreductase family protein (IorA), which can directly convert the indole-3-pyruvic acid pathway (IPA/IPyA) to IAA, was also found. Furthermore, the phytoene/squalene synthase family protein (PSY) was identified, which is involved in the synthesis of ABA (**Supplementary Table S4**). The phytase, exopolyphosphatase and glucose 1-dehydrogenase genes were found in T3E, which provides adequate amounts of P for their own metabolism and that of their host plant (**Supplementary Table S5**). In the T3E strain, the five enzymes Carbon-nitrogen hydrolase family protein, P-II family nitrogen regulator, Nitrogen regulation protein NR(I), Nitroreductase, and Nitronate monooxygenase were identified, which are directly or indirectly involved in microbial nitrogen fixation, contributing to the plant’s acquisition of nitrogen from microbial nitrogen fixation. (**Supplementary Table S6**).

### Effects of T3E colonization on soil physicochemical properties and enzymatic activities

**Figure 7:**
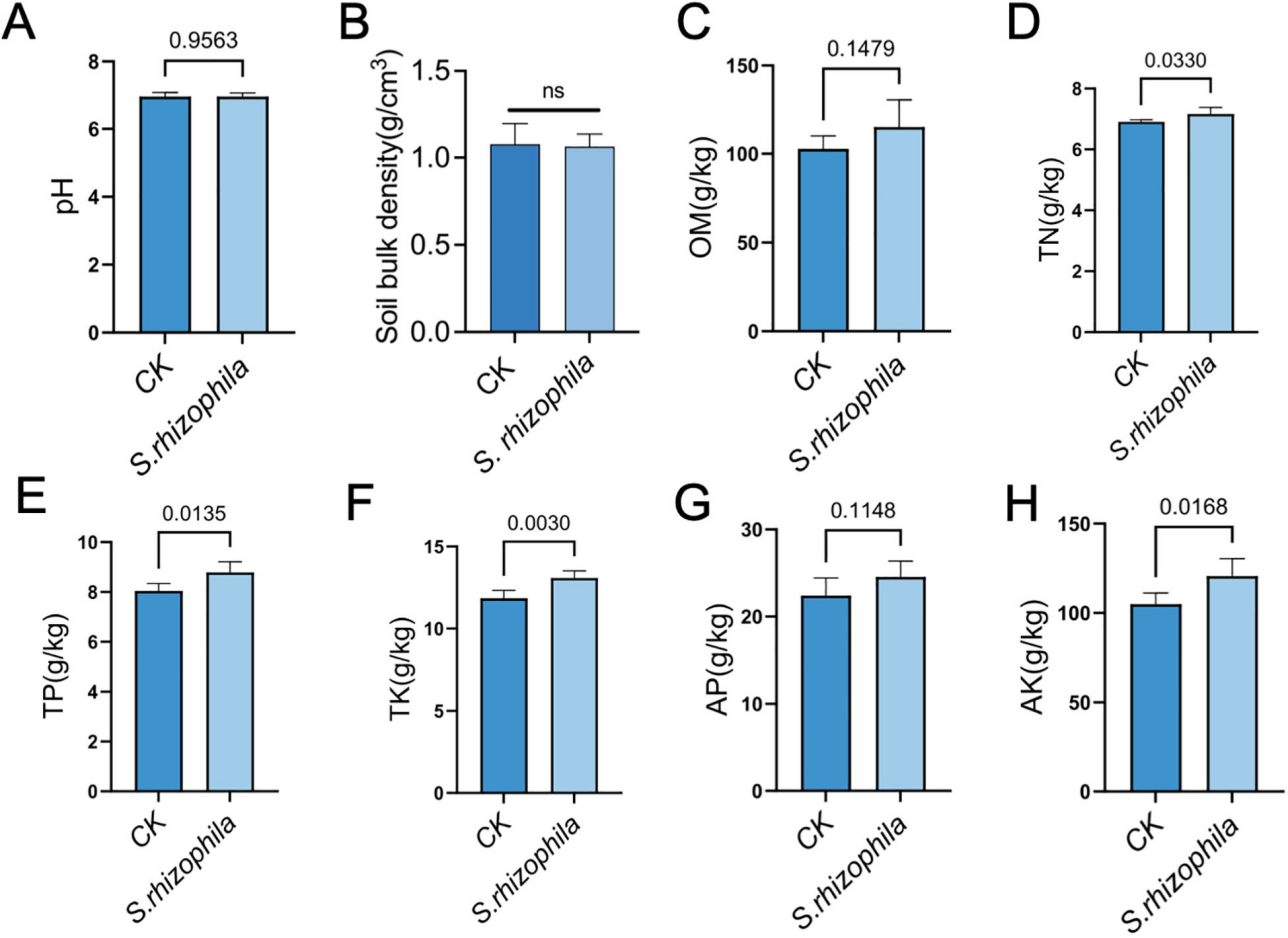
Effects of *S. rhizophila* T3E on soil physicochemical properties and enzyme activities. Soil from tomato rhizospheres was analyzed after treatment with T3E or sterile control (CK). **(A)** pH, **(B)** Soil bulk density, **(C)** organic matter (OM), **(D)** total nitrogen (TN) content, **(E)** total phosphorus (TP), **(F)** total potassium (TK), **(G)** available phosphorus (AP), **(H)** available potassium (AK). The error bars indicate the ± SDs of the means. Statistical significance was determined by two-sided Student’s t-test. ns, not significant. p values are indicated. Different letters represent statistically significant differences.

T3E treatment led to a pronounced enrichment of TN (7.17 ± 0.22 vs. 6.91 ± 0.07), TP (8.78 ± 10.43 vs. 8.04 ± 0.30), TK (13.08 ± 0.44 vs. 11.84 ± 0.49), and AK (120.63 ± 9.83 vs. 104.93 ± 6.26) compared with the control group (p < 0.05). However, soil pH (6.96 ± 0.10 vs. 6.96 ± 0.12), Soil bulk density (1.11 ± 0.18 vs. 1.09 ± 0.13), OM (115.06 ± 15.42 vs. 102.87 ± 7.20) and AP (24.56 ± 1.81 vs. 22.41 ± 2.03) were statistically indistinguishable from the control (*p* > 0.05) (**Figures 7A–H**).

**Figure 8:**
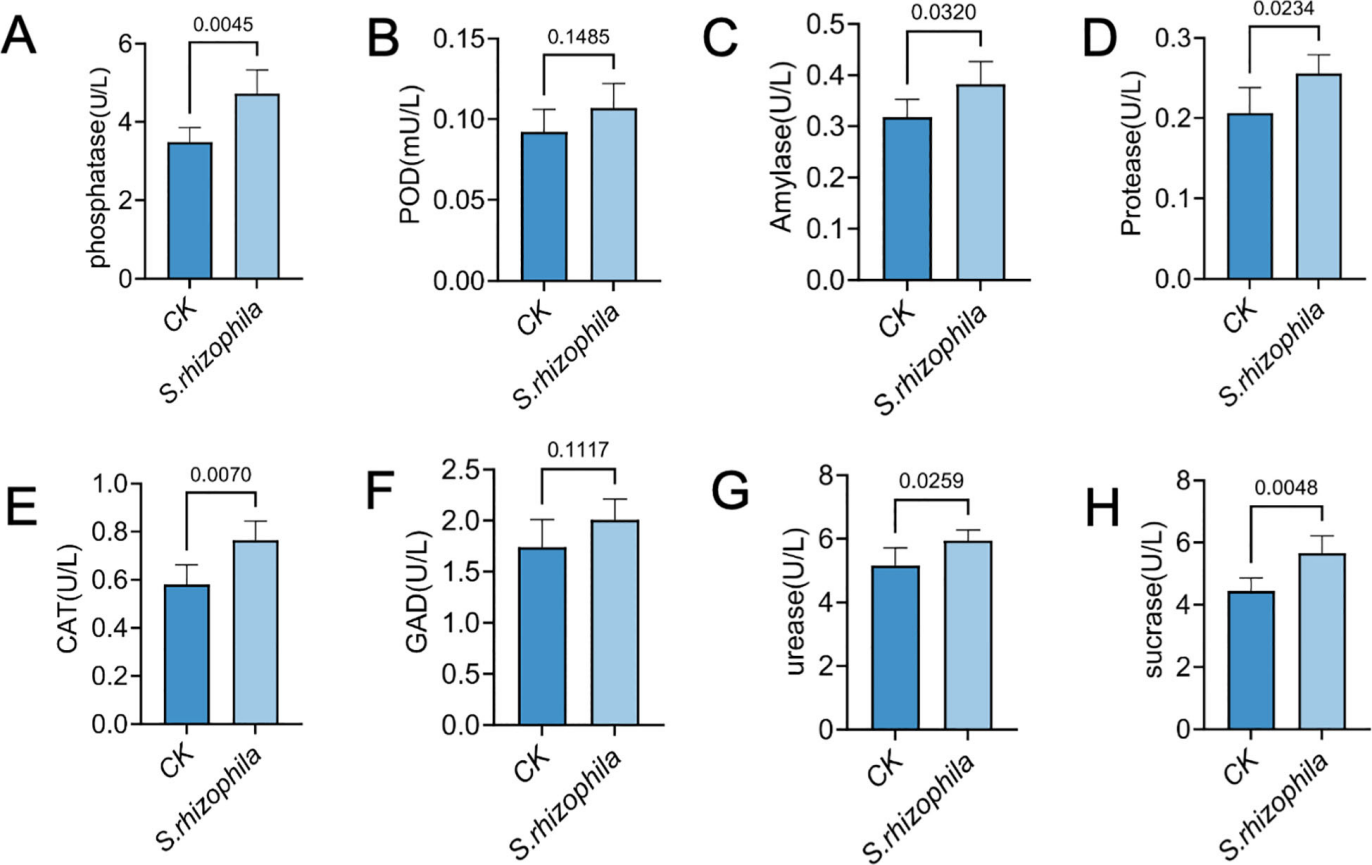
Soil enzyme activities in response to *S. rhizophila* T3E inoculation. Activities of eight functionally diverse enzymes were quantified in tomato rhizosphere soils following T3E or control (CK) treatment. **(A)** phosphatase activity, **(B)** peroxidase (POD) activity, **(C)** amylase activity, **(D)** protease activity, **(E)** (CAT) activity. **(F)** glutamic acid decarboxylase (GAD), **(G)** urease, **(H)** sucrase in T3E-treated soil. The error bars indicate the ± SDs of the means. Statistical significance was determined by two-sided Student’s t-test. ns, not significant. p values are indicated. Different letters represent statistically significant differences.

Concurrently, enzymatic profiling revealed significant upregulation of phosphatase (4.72 ± 0.61 vs. 3.48 ± 0.37), amylase (0.38 ± 0.04 vs. 0.32 ± 0.03), protease (0.26 ± 0.02 vs. 0.21 ± 0.03), CAT (0.76 ± 0.08 vs. 0.58 ± 0.08), urease (5.95 ± 0.33 vs. 5.16 ± 0.55) and sucrase (5.65 ± 0.56 vs. 4.44 ± 0.42) activities under LP0308 inoculation (p < 0.05) compared with the control group (*p* < 0.05), whereas POD (0.11 ± 0.02 vs. 0.09 ± 0.01) and GAD (2.01 ± 0.20 vs. 1.74 ± 0.27) did not differ statistically from the control (p = 0.76 and 0.25, respectively) (**Figures 8A–H**).

## Discussion

PGPR are considered indispensable components of rhizosphere engineering due to their capacity to improve plant growth and development. In the present study, a *S. rhizophila* strain T3E isolated from the tomato rhizosphere was characterized to validate its plant growth–promoting potential.

SEM micrographs clearly reveal the formation of OMVs on the bacterial surface. OMVs are spherical protrusions of the outer membrane that bud spontaneously from the bacterial surface under a variety of environmental conditions. Studies have revealed that OMVs mediate the targeted delivery of growth-promoting enzymes and elicitors to plant roots, while modulating bacterial defenses to preserve microbial community stability (Li et al., 2023). For example, proteolytic enzymes and antibiotic-active secondary metabolites (citilin A, myxovirescin A, myxalamide, and myxamine) detected in Myxococcus xanthus OMVs contribute to the killing of microbial prey (Berleman et al., 2014; Schwechheimer and Kuehn, 2015).

Genomic analysis revealed that the T3E strain harbors diverse genes encoding CAZymes, hormone biosynthetic pathways, nutrient acquisition systems, and nitrogen fixation–related enzymes, highlighting its multifaceted mechanisms in promoting plant growth. These findings demonstrate that T3E functions as a potent PGPR. Consistently, tomato bioassays further confirmed that T3E significantly promotes seed germination and seedling growth by enhancing root development and elevating IAA and ABA levels. PGPR are beneficial microorganisms that establish symbiotic relationships with plants (Wang et al., 2024). Species of *Azospirillum*, *Pseudomonas*, *Alcaligenes*, *Azotobacter*, *Klebsiella*, *Arthrobacter*, *Enterobacter*, *Bacillus*, *Lysobacter*, and *Serratia* promote plant growth as PGPR by boosting plant immunity, improving soil fertility, and facilitating the assimilation of essential nutrients (Nephali et al., 2022; Khoso et al., 2024). For example, *Bacillus megaterium* NCT-2 significantly enhanced nitrate removal from the soil and improved plant growth by colonizing the meristematic and elongation zones of the root tip as well as the roots (Chu et al., 2018). *S. rhizophila* SR80, isolated from the wheat rhizosphere, enhances both below− and above−ground plant growth and induces robust disease resistance by strengthening plant defense responses (Liu et al., 2021). Importantly, the levels of endogenous IAA and ABA in tomato seedlings significantly increased after T3E inoculation. Similar results were observed, the strain of *Trichoderma longibrachiatum* T6 effectively promoted wheat growth and enhanced plant tolerance to NaCl stress through the increased IAA production (Zhang et al., 2019). IAA is acknowledged as a key mechanism through which PGPR promote plant growth (Park et al., 2021). Over 80% of rhizosphere‐associated bacteria-such as those belonging to the genera *Azotobacter*, *Azospirillum*, *Staphylococcus*, *Pseudomonas* and *Enterobacter*-are capable of producing IAA (Park et al., 2017; Bag et al., 2022).Inoculation of *Arabidopsis thaliana* with *Azospirillum brasilense* Sp245 elevates ABA levels, with the increase most pronounced under osmotic stress (Khoso et al., 2024). Therefore, the IAA and ABA secreted by T3E may contribute to its growth-promoting effect.

The successful colonization of T3E led to drastically increased expression of encoding nutritional/metabolic factor(*pyrB*), motility(*flmH*), adherence(*pilR*) and biofilm(*bopD*) proteins in the rhizosphere soil on a genome-wide scale. Aspartate aminotransferases (*pyrB*) are indispensable for plant growth, reproduction, development, and defense and function as key enzymes in the biosynthesis of essential amino acids including lysine, isoleucine, methionine, threonine, tyrosine, and phenylalanine (de la Torre et al., 2014). Additionally, the short‐chain dehydrogenase/reductase *FlmH* has been shown to play a crucial role in plant peroxisomes by catalyzing the conversion of indole-3-butyric acid (IBA) to IAA. This enzymatic activity is essential for plant reproduction and seed development (Zhang et al., 2023). In *P. aeruginosa*, the *PilR* two-component system regulates both twitching and swimming motilities, enabling the bacteria to attach to host cells (but not to mucin), translocate along the cell surface, and form biofilms (Kilmury and Burrows, 2018). Briefly, the core T3E gene increases bacterial adherence to the host, preventing cells from being flushed off root surfaces and thereby promoting successful rhizosphere colonization. Furthermore, T3E actively secretes the microbial secondary metabolites, which may enhance its competitive fitness in soil-plant system, such as RiPP-like, lanthipeptide-class-ii and arylpolyene. Studies on RiPP-like peptides have demonstrated their ability to bind the peptidoglycan precursor lipid II, thereby inhibiting cell-wall assembly and inducing bacterial cell death (Asamizu, 2025; Smith et al., 2025). We propose that T3E indirectly enhances its soil colonization by secreting RiPP-like peptides to outcompete other microorganisms for ecological niches.

Inoculation of T3E into soil significantly altered tomato root immune gene expression and enzyme activities. We found that T3E treatment upregulated several root growth-related genes, including *EAT2*, *LAX2*, *GTS1*, and *GRFs*. *EAT2*, an evolutionarily conserved member of the AP2/ethylene-responsive factor (*ERF*) plant family, regulates various developmental and stress response pathways, enhancing tolerance to both biotic and abiotic stresses (Diaz-Manzano et al., 2018). In addition, molecular cloning of *LAX2* reveals that it encodes a nucleoprotein with a plant-specific conserved domain and regulates aboveground branching in rice during development (Tabuchi et al., 2011). In our study, the activity of SOD, and the contents of glycine betaine and soluble sugars in tomato roots inoculated with T3E were significantly increased compared to the control. It is well known that SOD, POD, CAT, and APX are four crucial enzymes in the plant defense system (Chen et al., 2024a). Research has shown that SOD, one of the most important plant antioxidant enzymes, mitigates environmental stresses by converting ROS into H_2_O_2_, which is subsequently broken down into water and oxygen by other enzymes (Huang et al., 2023). Glycine betaine has been identified as a crucial osmoprotectant that significantly contributes to bacterial adaptation and survival under diverse environmental stresses (Boysen et al., 2021). Moreover, soluble sugars and organic acids are mainly decisive factor of flavor quality in tomato (Shen et al., 2020).

Additionally, T3E markedly enriched soil nutrients and upregulated major enzymatic activities processes, supporting its potential to improve rhizosphere fertility. We found that T3E treatment led to a pronounced enrichment of TN, TP, TK and AK compared with the control group. For example, *B.s aryabhattai* (MB) significantly enhanced soil available potassium, leading to a 7.62-12.7% increase in tomato yield and improvements in fruit quality, as evidenced by 13.7-17.1% higher soluble sugar and 8.46-8.93% higher vitamin C contents (Guo et al., 2024). Lee et al. demonstrated that soil application of *Rhodopseudomonas palustris* PS3 significantly enhanced nutrient availability (from 35% to 56%) and elevated internal nitrogen accumulation in tomatoes under organic cultivation (Lee et al., 2022). In addition, the contents of TPTKAP and AK were identified as key factors influencing bacterial community diversity (Deng et al., 2024).

In addition to nutrients, we also found that the activities of some soil enzymes, such as phosphatase, CAT and sucrase, were significantly increased. Research found that the application of *Sinorhizobium meliloti* CCNWSX0020 significantly improved soil microbial attributes, notably enhancing alkaline phosphatase activity and microbial biomass, which collectively contributed to the promotion of alfalfa growth (Duan et al., 2021). Phosphatases are key enzymes that hydrolyze organic phosphorus compounds, contributing significantly to soil quality improvement (Yang et al., 2017; Wang et al., 2020). Therefore, we hypothesize that T3E proliferates within the soil microbiome, consequently facilitating nutrient turnover and availability. Pseudomonas sp. PA-1, a rhizosphere and endophytic associated strain from lettuce, markedly elevated soil sucrase activity in the root zone, with an observed increase of up to 247% (Wen et al., 2023).

## Conclusion

These findings highlight the multifaceted role of Stenotrophomonas rhizophila T3E as a promising PGPR with potential applications in sustainable agriculture. By modulating root development, activating plant immune responses, enhancing soil nutrient availability, and stimulating beneficial enzymatic activities, T3E demonstrates strong potential to reduce reliance on chemical fertilizers and improve crop productivity and quality. Future studies should explore the field-level efficacy of T3E under diverse environmental conditions, as well as its synergistic interactions with native soil microbiota, to fully harness its capabilities for rhizosphere engineering and eco-friendly crop management strategies.

## Data Availability

The datasets presented in this study can be found in online repositories. The names of the repository/repositories and accession number(s) can be found below: https://www.ncbi.nlm.nih.gov/, PRJNA1242732.

## References

[B1] Asamizu S. (2025). Recent advances in discovery and biosynthesis of ribosomally synthesized and post-translationally modified peptides (RiPP)-derived lipopeptides. Nat. Prod Rep. 3, 1–31. doi: 10.1039/d5np00042d, PMID: 40665846

[B2] Bag S. Mondal A. Banik A. (2022). Exploring tea (*Camellia sinensis*) microbiome: Insights into the functional characteristics and their impact on tea growth promotion. Microbiol. Res. 254, 126890. doi: 10.1016/j.micres.2021.126890, PMID: 34689100

[B3] Bao S. D. (2000). Soil and agricultural chemistry analysis (Beijing: China Agriculture Press).

[B4] Barringer S. (2004). “Canned tomatoes: Production and storage,” in Handbook of vegeta ble preservation and processing. Eds. Hui Y. H. Ghazala S. Graham D. M. Murrell K. D. Nip W. (CRC Press, New York, NY, USA), 109–120.

[B5] Benson G. (1999). Tandem repeats finder: a program to analyze DNA sequences. Nucleic Acids Res. 27, 573–580. doi: 10.1093/nar/27.2.573, PMID: 9862982 PMC148217

[B6] Berleman J. E. Allen S. Danielewicz M. A. Remis J. P. Gorur A. Cunha J.. (2014). The lethal cargo of *Myxococcus xanthus* outer membrane vesicles. Front. Microbiol. 5. doi: 10.3389/fmicb.2014.00474, PMID: 25250022 PMC4158809

[B7] Bhojiya A. A. Joshi H. Upadhyay S. K. Srivastava A. K. Pathak V. V. Pandey V. C.. (2022). Screening and Optimization of Zinc Removal Potential in *Pseudomonas aeruginosa*-HMR1 and its Plant Growth-Promoting Attributes. Bull. Environ. Contam Toxicol. 108, 468–477. doi: 10.1007/s00128-021-03232-5, PMID: 33860803

[B8] Blin K. Wolf T. Chevrette M. G. Lu X. Schwalen C. J. Kautsar S. A.. (2017). antiSMASH 4.0-improvements in chemistry prediction and gene cluster boundary identification. Nucleic Acids Res. 45, W36–W41. doi: 10.1093/nar/gkx319, PMID: 28460038 PMC5570095

[B9] Boysen A. K. Carlson L. T. Durham B. P. Groussman R. D. Aylward F. O. Ribalet F.. (2021). Particulate metabolites and transcripts reflect diel oscillations of microbial activity in the surface ocean. mSystems 6, e00896-20. doi: 10.1128/mSystems.00896-20, PMID: 33947808 PMC8269247

[B10] Chattaraj S. Samantaray A. Ganguly A. Thatoi H. (2025). Employing plant growth-promoting rhizobacteria for abiotic stress mitigation in plants: with a focus on drought stress. Discover Appl. Sci. 7, 68. doi: 10.1007/s42452-025-06468-6

[B11] Chauviat A. Abrouk D. Brothier E. Muller D. Meyer T. Favre-Bonte S. (2025). Genomic and phylogenetic re-assessment of the genus *Stenotrophomonas*: Description of *Stenotrophomonas thermophila* sp. nov., and the proposal of *Parastenotrophomonas gen.* Nov., *Pseudostenotrophomonas* gen. Nov., *Pedostenotrophomonas* gen. Nov., and *Allostenotrophomonas* gen. Nov. Syst. Appl. Microbiol. 48, 126630. doi: 10.1016/j.syapm.2025.126630, PMID: 40550196

[B12] Chen F. Ha X. Ma T. Ma H. (2024a). Comparative analysis of the physiological and transcriptomic profiles reveals alfalfa drought resistance mechanisms. BMC Plant Biol. 24, 954. doi: 10.1186/s12870-024-05671-8, PMID: 39394556 PMC11470740

[B13] Chen L. Liu Y. (2024b). The function of root exudates in the root colonization by beneficial soil rhizobacteria. Biol. (Basel). 13, 95. doi: 10.3390/biology13020095, PMID: 38392313 PMC10886372

[B14] Chu S. Zhang D. Zhi Y. Wang B. Chi C. P. Zhang D.. (2018). Enhanced removal of nitrate in the maize rhizosphere by plant growth-promoting *Bacillus megaterium* NCT-2, and its colonization pattern in response to nitrate. Chemosphere. 208, 316–324. doi: 10.1016/j.chemosphere.2018.05.189, PMID: 29883866

[B15] Creti R. Koch S. Fabretti F. Baldassarri L. Huebner J. (2006). Enterococcal colonization of the gastro-intestinal tract: role of biofilm and environmental oligosaccharides. BMC Microbiol. 6, 60. doi: 10.1186/1471-2180-6-60, PMID: 16834772 PMC1534043

[B16] De Kesel J. Conrath U. Flors V. Luna E. Mageroy M. H. Mauch-Mani B.. (2021). The induced resistance lexicon: do’s and don’ts. Trends Plant Sci. 26, 685–691. doi: 10.1016/j.tplants.2021.01.001, PMID: 33531282

[B17] de la Torre F. Canas R. A. Pascual M. B. Avila C. Canovas F. M. (2014). Plastidic aspartate aminotransferases and the biosynthesis of essential amino acids in plants. J. Exp. Bot. 65, 5527–5534. doi: 10.1093/jxb/eru240, PMID: 24902885

[B18] Deng Y. Kong W. Zhang X. Zhu Y. Xie T. Chen M.. (2024). Rhizosphere microbial community enrichment processes in healthy and diseased plants: implications of soil properties on biomarkers. Front. Microbiol. 15. doi: 10.3389/fmicb.2024.1333076, PMID: 38505554 PMC10949921

[B19] Diaz-Manzano F. E. Cabrera J. Ripoll J. J. Del Olmo I. Andres M. F. Silva A. C.. (2018). A role for the gene regulatory module microRNA172/TARGET OF EARLY ACTIVATION TAGGED 1/FLOWERING LOCUS T (miRNA172/TOE1/FT) in the feeding sites induced by Meloidogyne javanica in Arabidopsis thaliana. New Phytol. 217, 813–827. doi: 10.1111/nph.14839, PMID: 29105090 PMC5922426

[B20] Duan C. Mei Y. Wang Q. Wang Y. Li Q. Hong M.. (2021). Rhizobium inoculation enhances the resistance of alfalfa and microbial characteristics in copper-contaminated soil. Front. Microbiol. 12. doi: 10.3389/fmicb.2021.781831, PMID: 35095795 PMC8791600

[B21] Ercole T. G. Savi D. C. Adamoski D. Kava V. M. Hungria M. Galli-Terasawa L. V. (2021). Diversity of maize (*Zea mays* L.) rhizobacteria with potential to promote plant growth. Braz. J. Microbiol. 52, 1807–1823. doi: 10.1007/s42770-021-00596-y, PMID: 34458975 PMC8578223

[B22] Etesami H. Beattie G. A. (2018). Mining halophytes for plant growth-promoting halotolerant bacteria to enhance the salinity tolerance of non-halophytic crops. Front. Microbiol. 9. doi: 10.3389/fmicb.2018.00148, PMID: 29472908 PMC5809494

[B23] Favela A. (2021). Maize germplasm chronosequence shows crop breeding history impacts recruitment of the rhizosphere microbiome. ISME J. 15, 2454–2464. doi: 10.1038/s41396-021-00923-z, PMID: 33692487 PMC8319409

[B24] Feng Q. Luo Y. Liang M. Cao Y. Wang L. Liu C.. (2025). Rhizobacteria protective hydrogel to promote plant growth and adaption to acidic soil. Nat. Commun. 16, 1684. doi: 10.1038/s41467-025-56988-3, PMID: 39956869 PMC11830790

[B25] Fiodor A. Singh S. Pranaw K. (2021). The contrivance of plant growth promoting microbes to mitigate climate change impact in agriculture. Microorganisms. 9, 1841. doi: 10.3390/microorganisms9091841, PMID: 34576736 PMC8472176

[B26] Fu Y. Wang J. Su Z. Chen Q. Li J. Zhao J.. (2025). *Sinomonas gamaensis* NEAU-HV1 remodels the IAA14-ARF7/19 interaction to promote plant growth. New Phytol. 245, 2016–2037. doi: 10.1111/nph.20370, PMID: 39722601

[B27] Gachomo E. W. Jimenez-Lopez J. C. Baptiste L. J. Kotchoni S. O. (2014). GIGANTUS1 (GTS1), a member of Transducin/WD40 protein superfamily, controls seed germination, growth and biomass accumulation through ribosome-biogenesis protein interactions in. BMC Plant Biol. 14, 37. doi: 10.1186/1471-2229-14-37, PMID: 24467952 PMC3914372

[B28] Garin T. Brault A. Marais C. Briand M. Preveaux A. Bonneau S.. (2025). T6SS-mediated competition by *Stenotrophomonas rhizophila* shapes seed-borne bacterial communities and seed-to-seedling transmission dynamics. mSystems. 10, e0045725. doi: 10.1128/msystems.00457-25, PMID: 40667995 PMC12363174

[B29] Garin T. Brin C. Preveaux A. Brault A. Briand M. Simonin M.. (2024). The type VI secretion system of *Stenotrophomonas rhizophila* CFBP13503 limits the transmission of Xanthomonas campestris pv. campestris 8004 from radish seeds to seedlings. Mol. Plant Pathol. 25, e13412. doi: 10.1111/mpp.13412, PMID: 38279854 PMC10777753

[B30] Giordano M. Petropoulos S. A. Rouphael Y. (2021). Response and Defence Mechanisms of Vegeta ble Crops against Drought, Heat and Salinity Stress. Agriculture. 11, 463. doi: 10.3390/agriculture11050463

[B31] Guo X. Zhan N. Wang Z. Ding F. Yang Z. Cui X. (2024). Effects of the combination of *Bacillus aryabhattai* and calcium peroxide on soil silicon and potassium contents, the yield and quality of facility tomato. Arch. Agron. Soil Science. 70, 1–12. doi: 10.1080/03650340.2024.2427072

[B32] Hou S. Thiergart T. Vannier N. Mesny F. Ziegler J. Pickel B.. (2021). A microbiota-root-shoot circuit favours Arabidopsis growth over defence under suboptimal light. Nat. Plants. 7, 1078–1092. doi: 10.1038/s41477-021-00956-4, PMID: 34226690 PMC8367822

[B33] Huang X. Wu Y. Zhang S. Yang H. Wu W. Lyu L.. (2023). Changes in antioxidant substances and antioxidant enzyme activities in raspberry fruits at different developmental stages. Scientia Horticulturae. 321, 112314. doi: 10.1016/j.scienta.2023.112314

[B34] Jia H. Xu Y. Deng Y. Xie Y. Gao Z. Lang Z.. (2024). Key transcription factors regulate fruit ripening and metabolite accumulation in tomato. Plant Physiol. 195, 2256–2273. doi: 10.1093/plphys/kiae195, PMID: 38561990 PMC11213253

[B35] Khoso M. A. Wagan S. Alam I. Hussain A. Ali Q. Saha S.. (2024). Impact of plant growth-promoting rhizobacteria (PGPR) on plant nutrition and root characteristics: Current perspective. Plant Stress. 11, 100341. doi: 10.1016/j.stress.2023.100341

[B36] Kilmury S. L. N. Burrows L. L. (2018). The *pseudomonas aeruginosa* pilSR two-component system regulates both twitching and swimming motilities. mBio. 9, e01310-18. doi: 10.1128/mBio.01310-18, PMID: 30042200 PMC6058289

[B37] Kunal P. K. Kumawat K. C. Meena V. S. (2023). Editorial: Plant growth-promoting rhizobacteria (PGPR) and plant hormones: an approach for plant abiotic stress management and sustainable agriculture. Front. Microbiol. 14. doi: 10.3389/fmicb.2023.1285756, PMID: 37795303 PMC10546421

[B38] Lee S. K. Chiang M. S. Hseu Z. Y. Kuo C. H. Liu C. T. (2022). A photosynthetic bacterial inoculant exerts beneficial effects on the yield and quality of tomato and affects bacterial community structure in an organic field. Front. Microbiol. 13. doi: 10.3389/fmicb.2022.959080, PMID: 36118214 PMC9479686

[B39] Lee S. W. Cooksey D. A. (2000). Genes expressed in *Pseudomonas putida* during colonization of a plant-pathogenic fungus. Appl. Environ. Microbiol. 66, 2764–2772. doi: 10.1128/AEM.66.7.2764-2772.2000, PMID: 10877766 PMC92071

[B40] Lee D. Ellard M. Wanner L. A. Davis K. R. Douglas C. J. (1995). The Arabidopsis thaliana 4-coumarate:CoA ligase (4CL) gene: stress and developmentally regulated expression and nucleotide sequence of its cDNA. Plant Mol. Biol. 28, 871–884. doi: 10.1007/BF00042072, PMID: 7640359

[B41] Li P. Liu J. Saleem M. Li G. Luan L. Wu M.. (2022). Reduced chemodiversity suppresses rhizosphere microbiome functioning in the mono-cropped agroecosystems. Microbiome. 10, 108. doi: 10.1186/s40168-022-01287-y, PMID: 35841078 PMC9287909

[B42] Li Y. Wu J. Qiu X. Dong S. He J. Liu J.. (2023). Bacterial outer membrane vesicles-based therapeutic platform eradicates triple-negative breast tumor by combinational photodynamic/chemo-/immunotherapy. Bioact Mater. 20, 548–560. doi: 10.1016/j.bioactmat.2022.05.037, PMID: 35846843 PMC9253654

[B43] Lian H. Wang L. Ma N. Zhou C. M. Han L. Zhang T. Q.. (2021). Redundant and specific roles of individual MIR172 genes in plant development. PloS Biol. 19, e3001044. doi: 10.1371/journal.pbio.3001044, PMID: 33529193 PMC7853526

[B44] Liu Y. Gao J. Wang N. Li X. Fang N. Zhuang X. (2022). Diffusible signal factor enhances the saline-alkaline resistance and rhizosphere colonization of *Stenotrophomonas rhizophila* by coordinating optimal metabolism. Sci. Total Environ. 834, 155403. doi: 10.1016/j.scitotenv.2022.155403, PMID: 35469877

[B45] Liu H. Li J. Carvalhais L. C. Percy C. D. Prakash Verma J. Schenk P. M.. (2021). Evidence for the plant recruitment of beneficial microbes to suppress soil-borne pathogens. New Phytol. 229, 2873–2885. doi: 10.1111/nph.17057, PMID: 33131088

[B46] Lu Y. Zeng J. Liu Q. (2021). The rice miR396-GRF-GIF-SWI/SNF module: A player in GA signaling. Front. Plant Sci. 12. doi: 10.3389/fpls.2021.786641, PMID: 35087553 PMC8786800

[B47] McPherson M. R. Wang P. Marsh E. L. Mitchell R. B. Schachtman D. P. (2018). Isolation and analysis of microbial communities in soil, rhizosphere, and roots in perennial grass experiments. J. Vis. Exp. 24, 57932. doi: 10.3791/57932, PMID: 30102263 PMC6126543

[B48] Nephali L. Steenkamp P. Burgess K. Huyser J. Brand M. van der Hooft J. J. J.. (2022). Mass spectral molecular networking to profile the metabolome of biostimulant *bacillus* strains. Front. Plant Sci. 13. doi: 10.3389/fpls.2022.920963, PMID: 35755693 PMC9218640

[B49] Nichols K. A. Wright S. F. (2006). Carbon and nitrogen in operationally defined soil organic matter pools. Biol. Fertility Soils. 43, 215–220. doi: 10.1007/s00374-006-0097-2

[B50] Niu H. Nie Z. Long Y. Guo J. Tan J. Bi J.. (2023). Efficient pyridine biodegradation by *Stenotrophomonas maltophilia* J2: Degradation performance, mechanism, and immobilized application for wastewater. J. Hazard Mater. 459, 132220. doi: 10.1016/j.jhazmat.2023.132220, PMID: 37549577

[B51] Park S.-H. Elhiti M. Wang H. Xu A. Brown D. Wang A. (2017). Adventitious root formation of *in vitro* peach shoots is regulated by auxin and ethylene. Scientia Horticulturae. 226, 250–260. doi: 10.1016/j.scienta.2017.08.053

[B52] Park S. Kim A. L. Hong Y. K. Shin J. H. Joo S. H. (2021). A highly efficient auxin-producing bacterial strain and its effect on plant growth. J. Genet. Eng. Biotechnol. 19, 179. doi: 10.1186/s43141-021-00252-w, PMID: 34859356 PMC8639878

[B53] Schwechheimer C. Kuehn M. J. (2015). Outer-membrane vesicles from Gram-negative bacteria: biogenesis and functions. Nat. Rev. Microbiol. 13, 605–619. doi: 10.1038/nrmicro3525, PMID: 26373371 PMC5308417

[B54] Shen Q. Zhang S. Liu S. Chen J. Ma H. Cui Z.. (2020). Comparative transcriptome analysis provides insights into the seed germination in cotton in response to chilling stress. Int. J. Mol. Sci. 21, 2067. doi: 10.3390/ijms21062067, PMID: 32197292 PMC7139662

[B55] Shih Y. L. Wu C. M. Lu H. F. Li L. H. Lin Y. T. Yang T. C. (2022). Involvement of the hemP-hemA-smlt0796-smlt0797 Operon in Hemin Acquisition by *Stenotrophomonas maltophilia* . Microbiol. Spectr. 10, e0032122. doi: 10.1128/spectrum.00321-22, PMID: 35658602 PMC9241770

[B56] Singh R. P. Jha P. N. (2017). The PGPR Stenotrophomonas maltophilia SBP-9 Augments Resistance against Biotic and Abiotic Stress in Wheat Plants. Front. Microbiol. 8. doi: 10.3389/fmicb.2017.01945, PMID: 29062306 PMC5640710

[B57] Smith A. B. Ejindu R. C. Chekan J. R. (2025). Engineering RiPP pathways: strategies for generating complex bioactive peptides. Trends Biochem. Sci. 50, 495–507. doi: 10.1016/j.tibs.2025.04.001, PMID: 40335383

[B58] Tabuchi H. Zhang Y. Hattori S. Omae M. Shimizu-Sato S. Oikawa T.. (2011). LAX PANICLE2 of rice encodes a novel nuclear protein and regulates the formation of axillary meristems. Plant Cell. 23, 3276–3287. doi: 10.1105/tpc.111.088765, PMID: 21963665 PMC3203427

[B59] Wang N. Wang T. Chen Y. Wang M. Lu Q. Wang K.. (2024). Microbiome convergence enables siderophore-secreting-rhizobacteria to improve iron nutrition and yield of peanut intercropped with maize. Nat. Commun. 15, 839. doi: 10.1038/s41467-024-45207-0, PMID: 38287073 PMC10825131

[B60] Wang J. Wang X. Liu G. Wang G. Wu Y. Zhang C. (2020). Fencing as an effective approach for restoration of alpine meadows: Evidence from nutrient limitation of soil microbes. Geoderma. 363, 114148. doi: 10.1016/j.geoderma.2019.114148

[B61] Weiskopf S. R. Rubenstein M. A. Crozier L. G. Gaichas S. Griffis R. Halofsky J. E.. (2020). Climate change effects on biodiversity, ecosystems, ecosystem services, and natural resource management in the United States. Sci. Total Environ. 733, 137782. doi: 10.1016/j.scitotenv.2020.137782, PMID: 32209235

[B62] Wen Z. Liu Q. Yu C. Huang L. Liu Y. Xu S.. (2023). The difference between rhizosphere and endophytic bacteria on the safe cultivation of lettuce in cr-contaminated farmland. Toxics. 11, 371. doi: 10.3390/toxics11040371, PMID: 37112598 PMC10146757

[B63] Yang Y. Ji W. Qiao P. Fei N. Yang L. Guan W.. (2023). Acidovorax citrulli Type IV Pili PilR Interacts with PilS and Regulates the Expression of the pilA Gene. Horticulturae. 9, 1296. doi: 10.3390/horticulturae9121296

[B64] Yang W. Li P. Rensing C. Ni W. Xing S. (2017). Biomass, activity and structure of rhizosphere soil microbial community under different metallophytes in a mining site. Plant Soil. 434, 245–262. doi: 10.1007/s11104-017-3546-9

[B65] Youseif S. H. (2018). Genetic diversity of plant growth promoting rhizobacteria and their effects on the growth of maize plants under greenhouse conditions. Ann. Agric. Sci. 63, 25–35. doi: 10.1016/j.aoas.2018.04.002

[B66] Zhang S. Gan Y. Xu B. (2019). Mechanisms of the IAA and ACC-deaminase producing strain of *Trichoderma longibrachiatum* T6 in enhancing wheat seedling tolerance to NaCl stress. BMC Plant Biol. 19, 22. doi: 10.1186/s12870-018-1618-5, PMID: 30634903 PMC6330461

[B67] Zhang Y. Wang X. Wang X. Wang Y. Liu J. Wang S.. (2023). Bioinformatic analysis of short-chain dehydrogenase/reductase proteins in plant peroxisomes. Front. Plant Sci. 14. doi: 10.3389/fpls.2023.1180647, PMID: 37360717 PMC10288848

[B68] Zhao Y. Ding W. J. Xu L. Sun J. Q. (2024). A comprehensive comparative genomic analysis revealed that plant growth promoting traits are ubiquitous in strains of *Stenotrophomonas* . Front. Microbiol. 15. doi: 10.3389/fmicb.2024.1395477, PMID: 38817968 PMC11138164

[B69] Zhao P. Wang F. Deng Y. Zhong F. Tian P. Lin D.. (2022). Sly-miR159 regulates fruit morphology by modulating GA biosynthesis in tomato. Plant Biotechnol. J. 20, 833–845. doi: 10.1111/pbi.13762, PMID: 34882929 PMC9055814

[B70] Ziegler J. Voigtlander S. Schmidt J. Kramell R. Miersch O. Ammer C.. (2006). Comparative transcript and alkaloid profiling in Papaver species identifies a short chain dehydrogenase/reductase involved in morphine biosynthesis. Plant J. 48, 177–192. doi: 10.1111/j.1365-313X.2006.02860.x, PMID: 16968522

