# Supplementary material for: Isolation and characterization of Stenotrophomonas rhizophila T3E: a multifunctional rhizobacterium enhancing tomato growth and soil health
Source: Front Plant Sci. 2025 Oct 23;16:1692957. doi: 10.3389/fpls.2025.1692957 (PMC12589107; doi:10.3389/fpls.2025.1692957)
Supplement: Supplementary file 1 [file DataSheet1.docx]

Supplementary Material

**Supplementary Tables**

**Supplementary** **Table S1.** The following primers were used in this study.

| EAT 2  AP2a  LAX2  4CL  GTS1  GRFs  pyrB  flmH  pilR  bopD | CCTGCAGTAACATCTGAACA  TGAAGTTGAAGCTGCCAGA  CAATTTACTTATGGGCAACT  CCTCAGTTCGTACCTACGCA  TTGCTGCCGGGTGTAATTCT  ACCACGAAACTGCAGATGG  CGCACTGATCAACGCCGGC  AAGCAAACCTGATCAGCAT  AAGCAAACCTGATCAGCA  AACGGTTTCGCGGATCATC | CAGGCCACAACTACCACCTC  ATAATTGCAACTGCTGCAGC  TCCGATATGGTTTCTGGCCA  TGGATAGGCCCCAGCGCGGA  GATTGCCACTATTCAGTGGA  CCAATCTCGACGAGGGATCG  GCCGATGCGCTGATGATGCC  TGGGACGACGTGATGAAC  CCCAGCGCGTGCTGCCCG  GCGATTCGCGCAGCCCCGG |
| --- | --- | --- |

**
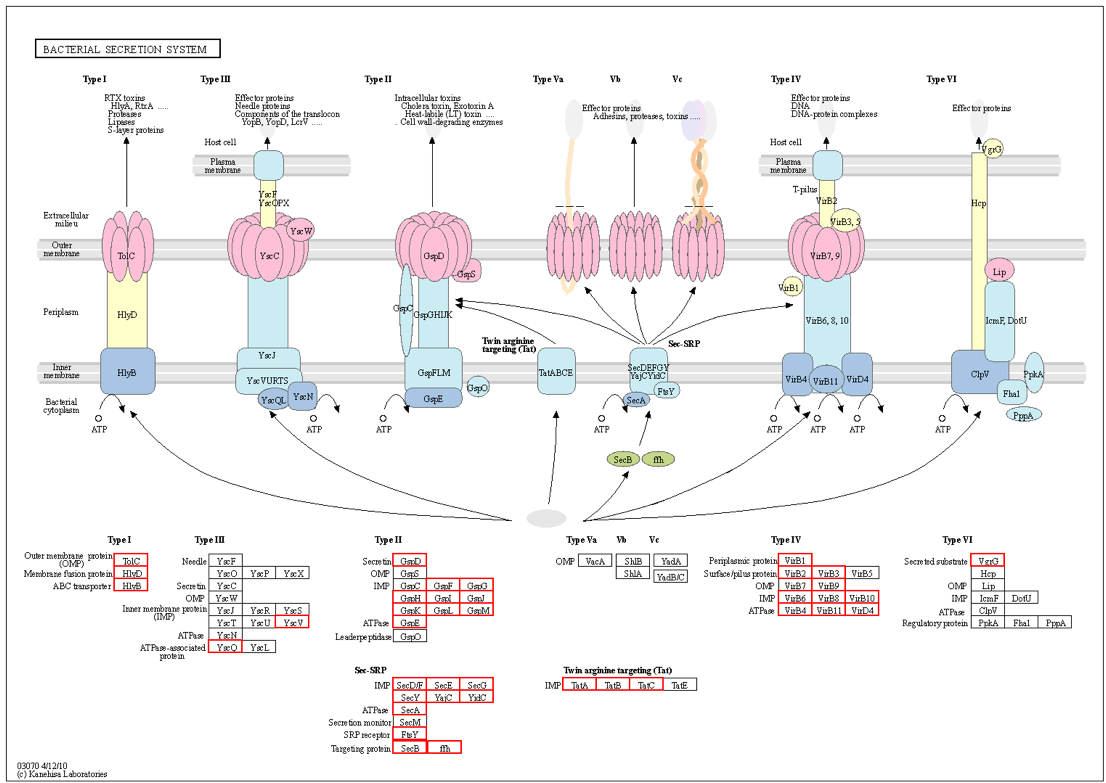
Supplementary Figures**

**Supplementary Figure S1.** T3E secretion systems encoded by the genome analyzed in this study.
